# Repetitive transcranial magnetic stimulation induced hypomanic symptoms in a woman with a history of electroconvulsive therapy induced mania:  a case report

**DOI:** 10.12688/f1000research.2-284.v1

**Published:** 2013-12-24

**Authors:** Noah S. Philip, S. Louisa Carpenter

**Affiliations:** 1 Center for Neurorestoration and Neurotechnology, Providence VA Medical Center, Department of Psychiatry & Human Behavior, Alpert Medical School, Brown University, Providence, RI, USA

## Abstract

Repetitive transcranial magnetic stimulation (rTMS) is a comparatively novel option for the treatment of major depressive disorder (MDD) and other psychiatric illnesses. Previous research has shown rTMS to be safe and without significant side effects compared to pharmacologic options. However, rare cases of rTMS-induced mania have been reported. This case report describes such an affective switch in a 52 year old female veteran with treatment-resistant MDD and a history of electroconvulsive therapy (ECT)-induced mania. Six treatments of rTMS were administered at 5 Hz for a total of 3000 pulses per day, when the patient began to display multiple hypomanic symptoms. These symptoms decreased after the termination of treatment and abated within a couple of days. In conclusion, caution should be used when administering rTMS to patients with a history of ECT-induced mania.

## Introduction

Repetitive transcranial magnetic stimulation (rTMS) is a US FDA-cleared neuromodulatory treatment for major depressive disorder (MDD). rTMS targets the dorsolateral prefrontal cortex, a brain region associated with atypical activation patterns in mood disorders ^1^. Both high (≥10 Hz, considered more excitatory) and lower (5 Hz) frequency rTMS has been shown to be efficacious in reducing depressive symptoms and is generally safe to use in MDD and other populations ^2– 5^. However, rare cases of rTMS-induced mania and hypomania have been reported in bipolar subjects ^6, 7^. The following case report documents 5 Hz rTMS-induced hypomanic symptoms in a patient with MDD but without a definitive history of bipolar disorder. Written informed consent for publication of their clinical details was obtained from the patient.

## Case report

Ms. A, a 52 year-old Caucasian veteran with MDD and post-traumatic stress disorder (PTSD), was referred to the rTMS clinic at her local Veterans Affairs hospital, due to continued depressive symptoms despite multiple unsuccessful prior pharmacologic interventions, in October of 2013. In her current major depressive episode, Ms. A had been treated with citalopram (40mg daily) and diazepam (10mg daily), with limited efficacy. On evaluation, Ms. A endorsed active depressive symptoms, including hypersomnia, intense feelings of sadness, weight loss, difficulty with decision-making, anhedonia, psychomotor retardation, anergia, and passive thoughts of death, without outright suicidal ideation. On measures of symptom severity, Ms. A reported scores consistent with a severe symptom burden on the Quick Inventory of Depressive symptoms, self-report (QIDS-SR = 19) ^8^, the Patient Health Questionnaire 9 (PHQ9 = 16) ^9^, and PTSD Checklist (PCL = 62) ^10^.

Regarding her past psychiatric history, Ms. A’s depressive and PTSD symptoms began during her childhood, when she experienced an extended period of reported sexual abuse from close family members and friends during the ages of 3–17. Her MDD and PTSD symptoms were exacerbated when she was sexually assaulted during military service at the age of 22. She still experiences active PTSD symptoms, including avoidance, hypervigilance, and marked response to triggers, on a daily basis. She reported a history of alcohol abuse but no substance abuse.

She had received multiple past interventions for her MDD and PTSD with limited efficacy or significant side effects. Previously trialled antidepressant included mono- and combination therapies of citalopram (up to 60mg daily), escitalopram (10mg daily), fluoxetine (up to 40mg daily), paroxetine (up to 20mg daily), sertraline (up to 50mg daily), bupropion (up to 450mg daily), mirtazapine (30mg at bedtime), nortriptyline (up to 40mg daily), duloxetine (20mg daily), and venlafaxine (up to 150mg daily). Previous augmentation regimens included treatment with lithium (up to 600mg daily), lamotrigine (up to 150mg daily), quetiapine (up to 200mg), risperidone (up to 1mg daily), olanzapine up to 15mg daily) and aripiprazole (up to 10mg daily). Additionally prazosin (up to 1mg daily), had been used for PTSD symptoms. She began a course of electroconvulsive therapy (ECT) 10 years ago, which was discontinued after 14 treatments due to an induced manic episode and subsequent hospitalization with pharmacotherapy for mood stabilization. Manic symptoms resolved after the termination of ECT treatment and since that time there have been no hypomanic or manic symptoms. She had not been on any pharmacotherapy for mood stability since 2003.

Past medical history included gastroesophageal reflux/Barrett’s esophagus, Graves’ disease and subsequent iatrogenic hypothyroidism, and chronic myelopathy. Associated current medications were levothyroxine 0.088mg, simvastatin 40mg, and sucralfate 500mg; all had been stable for >4 months prior to consultation. Results from a recent laboratory screening were within normal limits; most recent thyroid stimulating hormone level was 3.9 μIUnits/ml (within normal range). The physical and neurologic exams were consistent with her past medical history and revealed no acute changes.

After signing informed consent, rTMS was administered at 5 Hz for 4 sec, with an inter-train interval of 12 sec, over the left dorsolateral prefrontal cortex at 120% of the motor threshold for a total of 3000 pulses per session, delivered five days a week. This parameter choice reflected the current literature in addition to our previous clinical experience in which patients with significant anxiety often are unable to tolerate 10 Hz. After her fifth treatment Ms. A reported improved MDD and PTSD symptoms, with scores decreasing to 9, 11 and 43 for the QIDS, PHQ9 and PCL, respectively. After the sixth treatment, Ms. A arrived at the clinic displaying multiple hypomanic symptoms, including inflated self-esteem/grandiosity, hypermotoric behavior, pressured speech, and distractibility. She reported an increase in goal-directed behavior, namely shopping, and reduced sleep to 3–4 hours. She denied psychotic symptoms and risk-taking behaviors. She reported her “thoughts were racing” and she felt like "this could get worse and [she] would be in trouble." A diagnosis of rTMS-induced hypomanic symptoms was made. Treatment was suspended and symptoms were monitored. Hypomanic symptoms began decreasing in severity over the following 24 hours and continued to normalize over the next couple of days. A week post-termination of treatment, she reported stable mood and regular sleep of 6–8 hours per night for the previous 5 days, which was maintained at 2-weeks. Her scores on rating scales at one and two-weeks post-treatment reflected relatively retained improvements in mood and PTSD symptoms (QIDS-SR = 10; PHQ9 = 10; PCL = 50, and QIDS-SR = 10; PHQ9 = 11; PCL = 52 for one and two weeks, respectively). At one month post-treatment, there had been no evidence of hypomania since cessation of rTMS.

## Discussion

The strong temporal relationship between the presentation of symptoms and the course of treatment, as well as the patient’s history of ECT-induced mania, suggest that rTMS triggered Ms. A’s hypomanic symptoms. However, compared to previously reported cases ^6, 7^, the patient demonstrated no consistent symptoms of bipolar disorder prior to rTMS therapy.

The use of 5 Hz stimulation, compared to 10 Hz should also be noted. 5 Hz rTMS has been safely used in bipolar patients taking mood stabilizers ^3, 5, 11^. Lower frequency rTMS has been associated with an accelerated antidepressant effect as an add-on treatment to medication in MDD subjects compared to sham ^2^. Nonetheless, singular cases of rTMS-induced hypomania in bipolar patients have been reported for both 5 and 10 Hz parameters ^6, 7^.

We hypothesize that the increased treatment parameters (3000 pulses per day versus 1600 pulses per day in past 5 Hz literature) ^3, 5, 11^ and the aforementioned accelerating effect of rTMS treatment on antidepressants might have triggered the patient’s affective switch. Her history of ECT-induced mania, but not antidepressant-induced switching, suggests a mood regulatory system prone to severe shifts during neuromodulatory intervention. Unlike subjects in previous studies, Ms. A was not taking mood stabilizers that may act as a protective factor ^3, 5^. The presented case report therefore indicates that caution should be used when treating patients with rTMS who have a history of ECT-induced manic symptoms. Further study of patients with a history of affective switch with multiple neuromodulatory interventions is warranted, to better characterize a potentially at-risk population.

## Consent

Written informed consent for publication of their clinical details was obtained from the patient.
