# Supplementary material for: Repetitive transcranial magnetic stimulation induced hypomanic symptoms in a woman with a history of electroconvulsive therapy induced mania:  a case report
Source: F1000Res. 2013 Dec 24;2:284. [Version 1] doi: 10.12688/f1000research.2-284.v1 (PMC3954166; doi:10.12688/f1000research.2-284.v1)
Supplement: Peer review history

# Peer review: Referee response for version 1

**Published:** 2014-02-18

**Authors:** Martijn Arns

**Affiliations:** 1 Department of Experimental Psychology, Utrecht University, Utrecht, Netherlands

The authors describe an interesting case study of rTMS-induced hypomanic symptoms. It is important that such cases and information be published and made public, since it might also provide us more information about the working mechanism of rTMS, and in this case suggests some overlap between ECT and rTMS. The case is well written and clearly presented.

I have read this submission. I believe that I have an appropriate level of expertise to confirm that it is of an acceptable scientific standard.

# Peer review: Referee response for version 1

**Published:** 2014-01-06

**Authors:** Anne Weigand

**Affiliations:** 1 Berenson-Allen Center for Noninvasive Brain Stimulation, Beth Israel Deaconess Medical Center, Boston, MA, USA

The authors present an interesting case of rTMS-induced hypomanic symptoms in a 52 year old woman with treatment-resistant MDD and PTSD. Importantly, the patient had a history of a manic episode induced by a course of ECT 10 years ago. No hypomanic or manic symptoms have been reported after ECT termination and the patient had not been taking mood stabilizers since 2003.

To date, rare cases of affective switches triggered by rTMS have been reported in bipolar subjects. This important case report indicates that further caution is needed when treating patients with rTMS who have a history of ECT-induced mania.

I have read this submission. I believe that I have an appropriate level of expertise to confirm that it is of an acceptable scientific standard.
